# Erythema Ab Igne: Toasted Skin Syndrome as a Cutaneous Marker of Chronic Pain

**DOI:** 10.7759/cureus.86243

**Published:** 2025-06-17

**Authors:** Nuha Mahmood, Alana Pinheiro Alves, Thomas A Melgar

**Affiliations:** 1 Internal Medicine, Western Michigan University Homer Stryker M.D. School of Medicine, Kalamazoo, USA; 2 Infectious Disease, Henry Ford Health, Detroit, USA

**Keywords:** chronic abdominal pain, chronic cholecystitis, chronic pain, erythema ab igne, reticular pattern, toasted skin syndrome

## Abstract

Erythema ab igne (EAI) is a rare skin reaction caused by prolonged exposure to low-level heat, resulting in a reticular pattern of localized erythema and hyperpigmentation. This condition carries a potential risk of transformation into cutaneous malignancy. We present the case of a 38-year-old woman who developed a reticular-patterned rash on her abdomen. She had been using hot water bags to alleviate severe abdominal pain associated with chronic cholecystitis, which led to the development of hyperpigmented, mottled, erythematous skin changes across her entire abdomen. After discontinuing heat application, her hyperpigmentation significantly decreased, although the skin changes persisted years later.

## Introduction

Erythema ab igne (EAI) is a rare dermatological condition caused by chronic exposure to low-intensity heat or infrared radiation [1]. The term, which means "redness from fire," refers to its characteristic clinical appearance. Often called toasted skin syndrome, EAI presents as a non-blanching rash with reticular hyperpigmentation and mottled erythema localized to the heat-exposed area [2]. The condition typically arises from repeated exposure to heat sources that are not hot enough to burn the skin but are sufficient to cause cumulative thermal damage over time. Historically, EAI was linked to open fires or coal stoves; however, modern cases more commonly result from heating pads, laptop computers, hot water bottles, and other heated devices. The heat range that induces EAI is generally between 43°C and 47°C (109.4-116.6°F), applied repeatedly over weeks to months [3]. Although rare, the condition is more prevalent in individuals with chronic pain who frequently use heat therapy for pain relief.

We present a unique case of severe EAI secondary to chronic pain in a patient with delayed surgical intervention.

## Case presentation

A 38-year-old woman presented to the clinic with concerns about a "rash" on her abdomen, noted three weeks after undergoing a laparoscopic cholecystectomy. Her past medical history was significant for Hashimoto's thyroid disease, metabolic dysfunction-associated steatohepatitis with cirrhosis, and cholelithiasis resulting in acute cholecystitis. Due to her comorbidities, she was not a suitable candidate for surgery, so a cholecystostomy tube was placed for nine months before she was eventually cleared for laparoscopic cholecystectomy.

**Figure 1:**
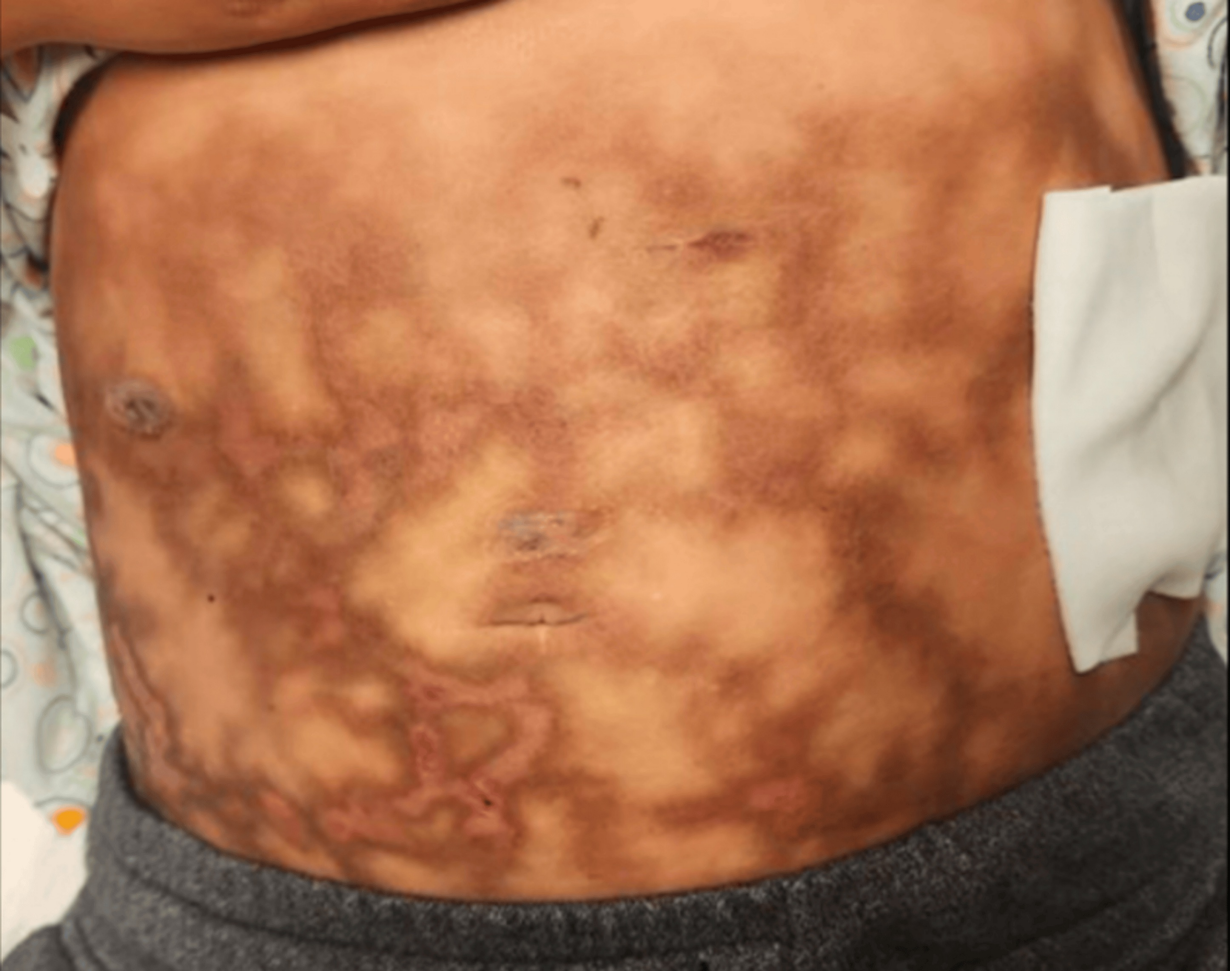
Initial presentation demonstrating reticular erythematous and hyperpigmented changes across the patient’s entire abdomen.

**Figure 2:**
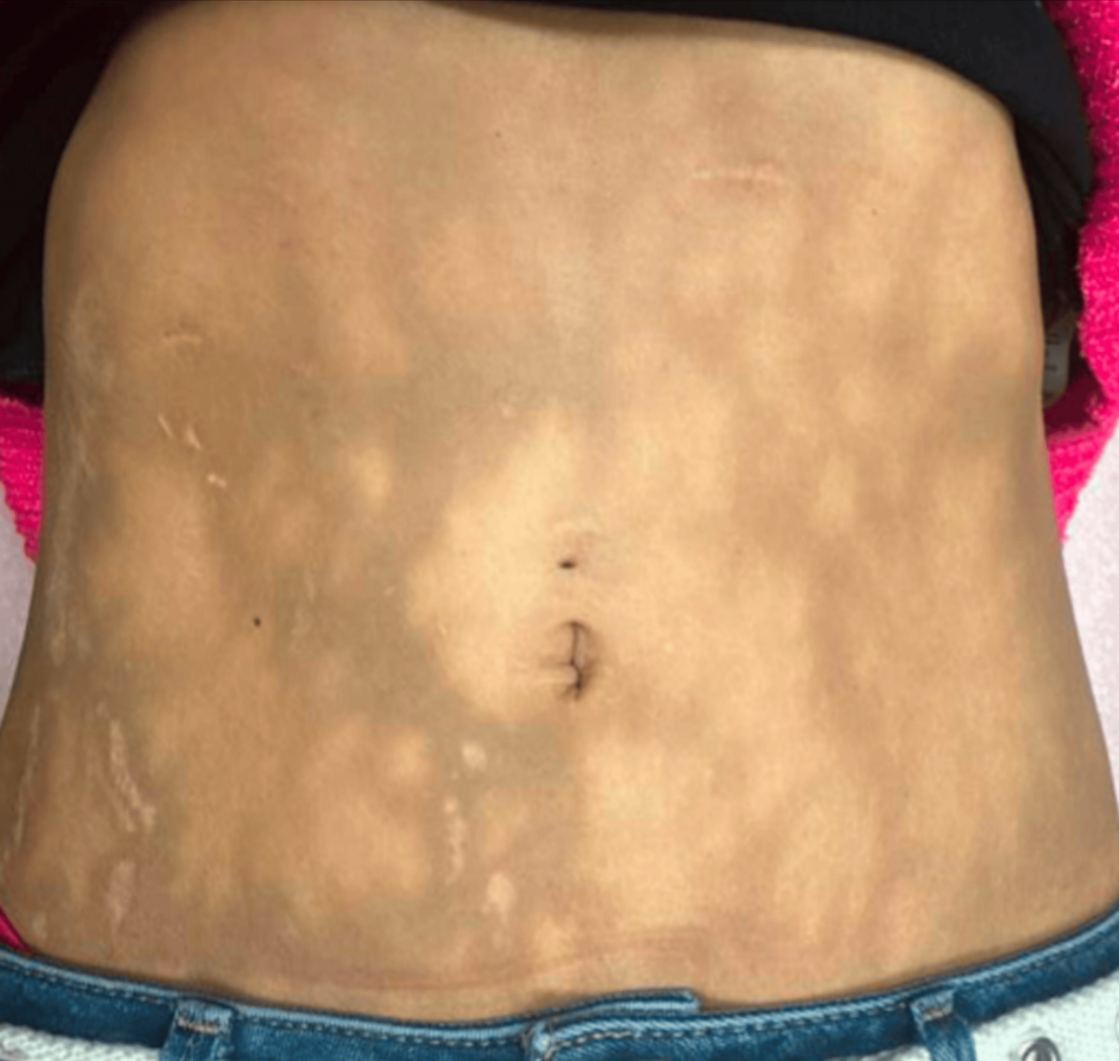
Two years post-cholecystectomy and discontinuation of hot water bottle use, showing persistent hyperpigmentation with markedly reduced erythema.

This delay in her surgical intervention resulted in severe, persistent, and chronic abdominal pain. To manage her symptoms, the patient regularly applied hot water bottles to her abdomen at night. Despite the prolonged exposure, she did not experience any sensation of burning. Over time, she developed a patterned skin discoloration, which she described as a “rash” (Figure 1). Physical examination revealed a well-demarcated, hyperpigmented, reticulated, mottled, and erythematous pattern across much of her abdomen, consistent with EAI. She denied exposure to other sources of heat or infrared light. She was prescribed topical hydroquinone 4% by her primary care physician, which she used for two months without noticeable improvement. On follow-up two years after her cholecystectomy, the patient no longer uses heat therapy for pain, and the skin changes have lightened in color (Figure 2). She denies any pain or burning sensation over the affected skin, although she continues to experience chronic right upper quadrant pain, for which she takes oxycodone 7.5 mg three times daily.

## Discussion

EAI is a cutaneous response to repeated exposure to infrared radiation or heat sources that are not intense enough to cause burns but can lead to cumulative skin damage over time. Several case reports have described patients with chronic pain who developed skin changes from the prolonged use of electric heating pads or other heat sources [1,2]. Our case is notable for the extensive area involved and the direct link between delayed surgical intervention for a painful condition and the prolonged use of hot water bottles. This highlights the need to recognize EAI as a physical manifestation of chronic pain management.

The pathophysiology of EAI is complex and remains incompletely understood, but several theories attempt to explain its characteristic reticular pattern and hyperpigmentation. One theory suggests that repeated heat exposure damages superficial blood vessels, leading to hemosiderin deposition in the skin and resulting in the typical vascular pattern and hyperpigmentation seen in chronic cases [3]. Another theory proposes that melanin is released from damaged elastic fibers in the skin, producing the dark red reticular rash [4]. Histopathological findings commonly include epidermal atrophy, dilated blood vessels, dermal hemosiderin deposits, and elastosis in the dermis [5-8].

The clinical course of EAI varies depending on the duration and intensity of heat exposure. Most cases are benign and resolved after discontinuation of the heat source [9]. However, as demonstrated in our patient, skin changes can persist long after the exposure has ceased. In addition, EAI carries a potential risk of malignant transformation, which may occur decades after the initial skin changes. Reported malignancies include squamous cell carcinoma, Merkel cell carcinoma, and other cutaneous malignancies [10,11]. This underscores the importance of long-term monitoring, especially in patients with extensive or persistent lesions.

The mainstay of treatment for EAI is the removal of the heat source, as seen in our patient. In cases where epidermal atypia is present or suspected, topical 5-fluorouracil may be considered [12]. For persistent hyperpigmentation, treatments such as hydroquinone, tretinoin, and various laser therapies have been reported with variable success [13,14]. Our patient’s trial of hydroquinone reflects its limited efficacy in treating established EAI-related skin changes, as it offered no significant improvement.

## Conclusions

This case highlights the importance of educating patients about the potential risks associated with prolonged heat application and recognizing EAI as a dermatologic manifestation of chronic pain, typically presenting as a reticular erythematous and hyperpigmented pattern. The presence of EAI should prompt clinicians to reassess pain management strategies, including potential adjustments to analgesic regimens for uncontrolled pain. While most cases of EAI are benign and resolve once the heat source is removed, rare instances of malignant transformation underscore the need for clinical vigilance and, when appropriate, long-term follow-up.

## References

[1] Wipf AJ, Brown MR (2022). Malignant transformation of erythema ab igne. JAAD Case Rep.

[2] Wells A, Desai A, Rudnick EW, Motaparthi K (2021). Erythema ab igne with features resembling keratosis lichenoides chronica. J Cutan Pathol.

[3] Tan S, Bertucci V (2000). Erythema ab igne: an old condition new again. CMAJ.

[4] Fareedy SB, Rettew A, Karmacharya P, Jehangir A, Shaikh B, Pathak R (2015). Erythema ab igne secondary to repeated heating pad use: an image case. J Community Hosp Intern Med Perspect.

[5] Ly V, Vandruff JE, Fashner J (2021). Erythema ab igne: toasted skin syndrome. HCA Healthc J Med.

[6] Milchak M, Smucker J, Chung CG, Seiverling EV (2016). Erythema ab igne due to heating pad use: a case report and review of clinical presentation, prevention, and complications. Case Rep Med.

[7] Finlayson GR, Sams WM Jr, Smith JG Jr (1966). Erythema ab igne: a histopathological study. J Invest Dermatol.

[8] Cavallari V, Cicciarello R, Torre V (2001). Chronic heat-induced skin lesions (erythema ab igne): ultrastructural studies. Ultrastruct Pathol.

[9] Miller K, Hunt R, Chu J, Meehan S, Stein J (2011). Erythema ab igne. Dermatol Online J.

[10] Sigmon JR, Cantrell J, Teague D, Sangueza O, Sheehan DJ (2013). Poorly differentiated carcinoma arising in the setting of erythema ab igne. Am J Dermatopathol.

[11] Jones CS, Tyring SK, Lee PC, Fine JD (1988). Development of neuroendocrine (Merkel cell) carcinoma mixed with squamous cell carcinoma in erythema ab igne. Arch Dermatol.

[12] Sahl W, Taira JW (1992). Erythema ab igne: treatment with 5-florouracil cream. J Am Acad Dermatol.

[13] Pennitz A, Kinberger M, Valle GA, Passeron T, Nast A, Werner RN (2022). Self-applied topical interventions for melasma: a systematic review and meta-analysis of data from randomized, investigator-blinded clinical trials. Br J Dermatol.

[14] Kim HW, Kim EJ, Park HC, Ko JY, Ro YS, Kim JE (2014). Erythema ab igne successfully treated with low fluenced 1,064-nm Q-switched neodymium-doped yttrium aluminum garnet laser. J Cosmet Laser Ther.

